# Supplementary material for: In Vitro Cell Proliferation and Migration Properties of Oral Mucosal Fibroblasts: A Comparative Study on the Effects of Cord Blood- and Peripheral Blood-Platelet Lysate
Source: Int J Mol Sci. 2023 Mar 17;24(6):5775. doi: 10.3390/ijms24065775 (PMC10058190; doi:10.3390/ijms24065775)
Supplement: Supplementary file 1 [file ijms-24-05775-s001.zip › ijms-2217087-supplementary.pdf]

Supplementary Materials: In Vitro Cell Proliferation and Migration Properties of Oral Mucosal Fibroblasts: A Comparative Study on the Effects of Cord Blood- and Peripheral Blood-Platelet Lysate

Arief Faisal Azmi [1], Mohammad Amirul Asyraff Mohd Yahya [1], Nur Ain Azhar [1], Norliwati Ibrahim [1], Norzana Abd Ghafar [2], Nur Azurah Abdul Ghani [3], Muhammad Aiman Mohd Nizar [1], Siti Salmiah Mohd Yunus [4], Tashveender Kaur Lakhbir Singh [1], Jia-Xian Law [5,*], Sook-Luan Ng [1,*]

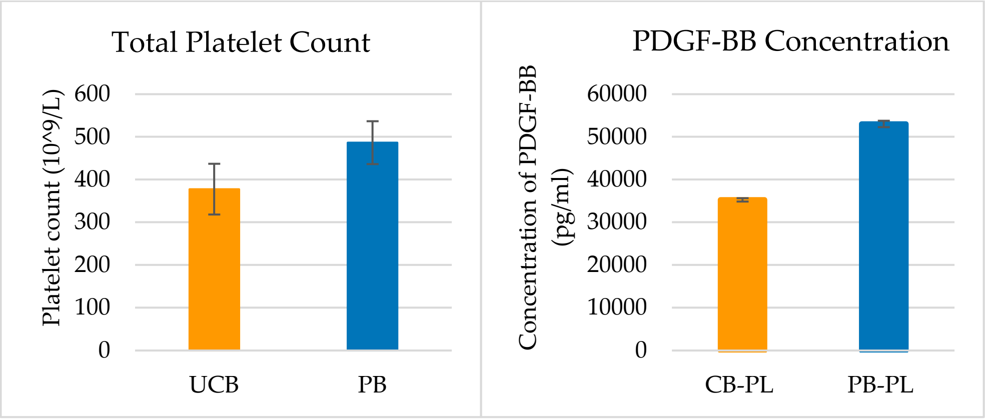

(a) **(b)**

Figure S1. Characterization of platelet lysates. (a) Total platelet concentration before performing freeze-thaw cycles to produce platelet lysates from umbilical cord blood (UCB) and peripheral blood (PB). (b) PDGF-BB concentration was quantified for pooled cord blood-platelet lysate (CB-PL) and peripheral blood-platelet lysate (PB-PL). All data were representative of six independent tests with n=6 by groups and means ± SEM.

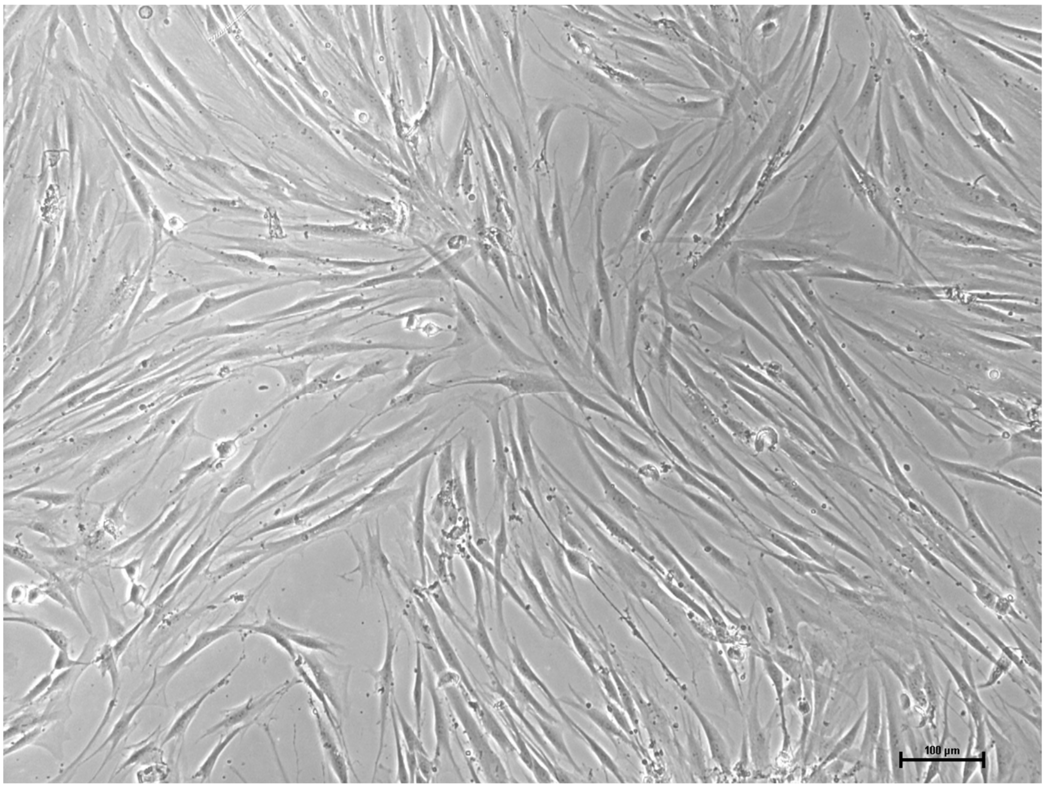

Figure S2. Cell morphology of human mucosal fibroblasts (HOMF) at passage 2 under 100× magnification. Cells showed fibroblastic features such as flat and spindle shape.

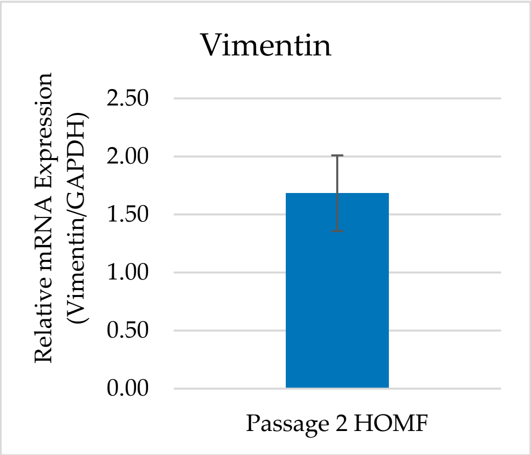

Figure S3. Relative mRNA expression of fibroblast marker (vimentin) in HOMF at passage 2. All data were representative of three independent tests with n=3 by groups and means ± SEM.

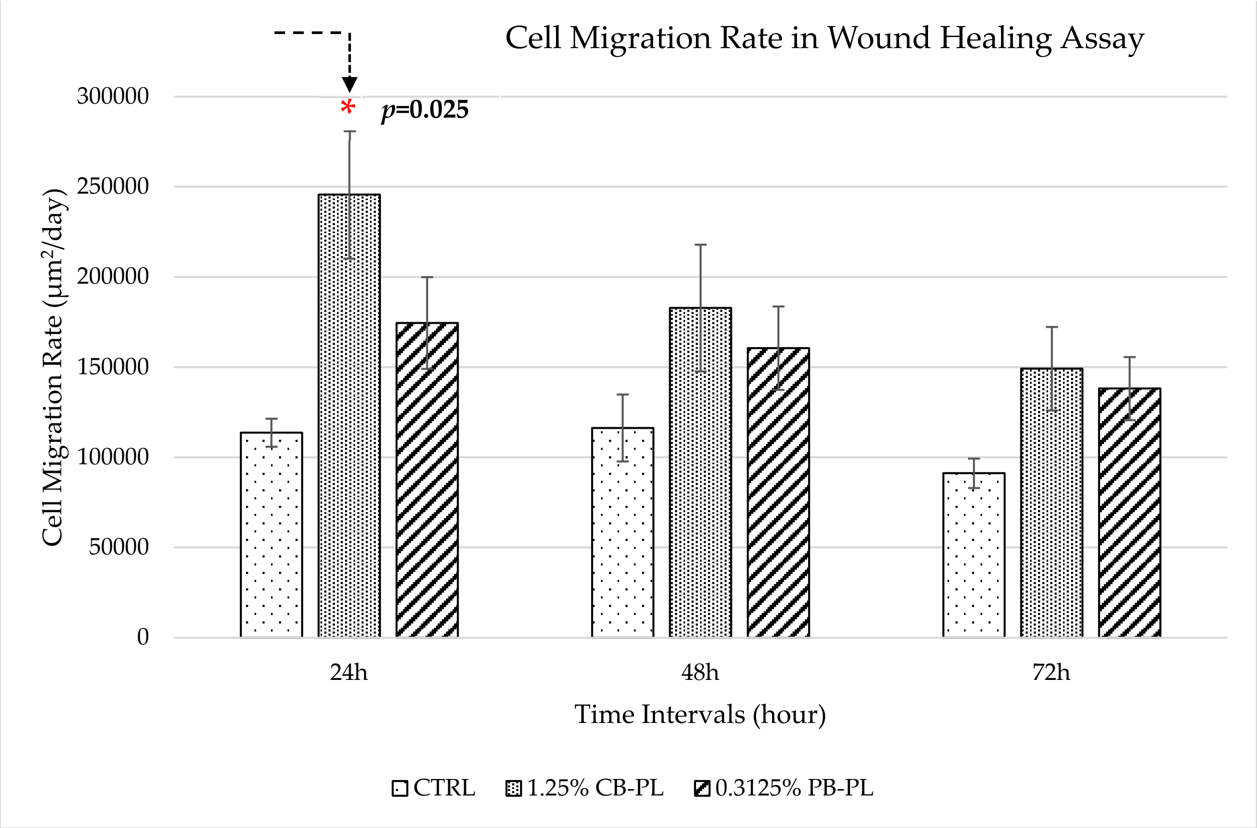

Figure S4. Cell migration rate of CTRL, 1.25% CB-PL, and 0.3125% PB-PL during wound healing assay. All data were representative of three independent tests with n=3 by groups and means ± SEM. $^*p < 0.05$ denoted the statistical significance of 1.25% CB-PL and 0.3125% PB-PL when compared to CTRL.
